# *N*-Glycosylation Is Important for *Halobacterium salinarum* Archaellin Expression, Archaellum Assembly and Cell Motility

**DOI:** 10.3389/fmicb.2019.01367

**Published:** 2019-06-18

**Authors:** Marianna Zaretsky, Cynthia L. Darnell, Amy K. Schmid, Jerry Eichler

**Affiliations:** ^1^Department of Life Sciences, Ben Gurion University of the Negev, Beersheba, Israel; ^2^Department of Biology, Duke University, Durham, NC, United States; ^3^Center for Genomics and Computational Biology, Duke University, Durham, NC, United States

**Keywords:** archaea, archaellin, archaellum, *Halobacterium salinarum*, motility, *N*-glycosylation

## Abstract

*Halobacterium salinarum* are halophilic archaea that display directional swimming in response to various environmental signals, including light, chemicals and oxygen. In *Hbt. salinarum*, the building blocks (archaellins) of the archaeal swimming apparatus (the archaellum) are *N*-glycosylated. However, the physiological importance of archaellin *N*-glycosylation remains unclear. Here, a tetrasaccharide comprising a hexose and three hexuronic acids decorating the five archaellins was characterized by mass spectrometry. Such analysis failed to detect sulfation of the hexuronic acids, in contrast to earlier reports. To better understand the physiological significance of *Hbt. salinarum* archaellin *N*-glycosylation, a strain deleted of *aglB*, encoding the archaeal oligosaccharyltransferase, was generated. In this Δ*aglB* strain, archaella were not detected and only low levels of archaellins were released into the medium, in contrast to what occurs with the parent strain. Mass spectrometry analysis of the archaellins in Δ*aglB* cultures did not detect *N*-glycosylation. Δ*aglB* cells also showed a slight growth defect and were impaired for motility. Quantitative real-time PCR analysis revealed dramatically reduced transcript levels of archaellin-encoding genes in the mutant strain, suggesting that *N*-glycosylation is important for archaellin transcription, with downstream effects on archaellum assembly and function. Control of AglB-dependent post-translational modification of archaellins could thus reflect a previously unrecognized route for regulating *Hbt. salinarum* motility.

## Introduction

In 1976, the surface (S)-layer glycoprotein from the hypersaline-adapted (halophilic) archaeon *Halobacterium salinarum* provided the first example of a glycosylated protein outside the Eukarya (Mescher and Strominger, 1976). Soon thereafter, *Hbt. salinarum* archaellins comprising the archaellum [the archaeal counterparts of bacterial flagellins and the flagellum, respectively (Jarrell and Albers, 2012)] were shown to be similarly modified (Wieland et al., 1985). Both the S-layer glycoprotein and archaellins were reported to be *N*-glycosylated by a tetrasaccharide comprising a glucose and three sulfated glucuronic acids initially assembled on a dolichol phosphate carrier (Lechner et al., 1985a; Wieland et al., 1985). After these initial reports and a series of biochemical studies aimed at delineating the *N*-glycosylation pathway involved (reviewed in Lechner and Wieland, 1989), published research on archaeal *N*-glycosylation was relatively limited until the mid-2000s, when a number of archaeal genome sequences became available and genetic tools for manipulating many of these species appeared. Presently, a considerable and growing body of data on archaeal *N*-glycosylation exists (reviewed in Jarrell et al., 2014), with most efforts in the field addressing S-layer glycoproteins and archaellins as reporters of this post-translational modification (Jarrell et al., 2010). Yet, despite the central role played by archaella in the directional taxis *Hbt. salinarum* displays in response to appropriate light, chemical, oxygen and other signals (Marwan et al., 1991), the importance of archaellin *N*-glycosylation in such directional swimming remains unclear.

Studies on other archaeal model species have revealed that archaellin *N*-glycosylation is important for proper archaellum assembly, function and cell motility across a wide variety of niches. These studies have largely focused on the methanogens *Methanococcus voltae* and *Methanococcus maripaludis* (Voisin et al., 2005; Chaban et al., 2006, 2009; Kelly et al., 2009; VanDyke et al., 2009; Jones et al., 2012; Ding et al., 2013, 2015, 2016; Siu et al., 2015), the halophile *Haloferax volcanii* (Tripepi et al., 2012) and the thermoacidophile *Sulfolobus acidocaldarius* (Meyer et al., 2015). In *M. maripaludis* and *Hfx. volcanii*, the absence of the archaeal oligosaccharyltransferase AglB disables archaellum assembly, despite comparable archaellin protein levels between the Δ*aglB* and parent strains (Abu-Qarn and Eichler, 2006; Chaban et al., 2006; VanDyke et al., 2009; Tripepi et al., 2012). In *S. acidocaldarius*, where *aglB* deletion is lethal, the elimination of archaellin *N*-glycosylation sites did not impact archaellum assembly or stability, yet compromised full motility (Meyer and Albers, 2014).

In the present study, *Hbt. salinarum* archaellin *N*-glycosylation and its importance were compared in a parent strain and in cells lacking *aglB*. Such efforts revealed the composition of the glycan decorating specific asparagine residues in *Hbt. salinarum* archaellins to differ from what was previously reported (Wieland et al., 1985). Moreover, the current study demonstrated the importance of *N*-glycosylation for archaellum assembly and cell motility in *Hbt. salinarum*, as well as for archaellin gene transcription and translation. These results thus suggest a novel role for *N*-glycosylation in *Hbt. salinarum*, namely the regulation of motility.

## Materials and Methods

### Cell Growth and Strains

*Halobacterium salinarum* NRC-1 (ATCC strain 700922) was used as the wild type strain background. The *Hbt. salinarum* Δ*ura3* strain (Peck et al., 2000) was used as the parent strain background for construction of the *Δura3ΔaglB* mutant (strain number AKS211). All strains were grown routinely in complete medium (CM) containing (per l) (250 g NaCl, 20 g MgSO_4_⋅7H_2_O, 3 g sodium citrate, 2 g KCl, 10 g peptone). The Δ*ura3* and *Δura3ΔaglB* (referred to as Δ*aglB* for brevity) cultures were supplemented with 50 μg/ml uracil to complement the uracil auxotrophy (Darnell et al., 2017). For evaluation of growth phenotypes, Δ*ura3* and Δ*aglB* cultures were prepared and grown in a Bioscreen C (Growth Curves USA) as previously described (Darnell et al., 2017), except that cultures were grown for 72 h with five biological replicates (inoculations with five independent colonies) and three technical replicates each. Growth rates were calculated as reported previously (Sharma et al., 2012; see also the GitHub repository associated with this publication for details^1^). Significance of the difference between growth rates of the parent vs. mutant strain was determined by Welch’s unpaired two-sided *t*-test across biological replicates (i.e., averaged technical replicates).

The *Hbt. salinarum* Δ*ura3* strain deleted of *aglB* (*VNG1068G*) was generated as previously reported using the standard double-crossover counter-selection method (Peck et al., 2000). Briefly, approximately 500 bp of flanking regions upstream and downstream of the *aglB* gene were PCR amplified (primers used are listed in Table 1) and inserted into the HindIII restriction site of plasmid pNBK07 (Wilbanks et al., 2012) by isothermal assembly (Gibson et al., 2009) to create plasmid pAKS140. Following Sanger sequencing, plasmid pAKS140 was introduced into the Δ*ura3* strain and selected on CM plates (CM medium with 20 g/l agar) containing mevinolin (10 μg/ml). The resulting merodiploid strains were then counter-selected on plates containing 5-fluoroorotic acid (300 μg/ml) and uracil to remove the integrated plasmid, yielding the unmarked Δ*aglB* strain, termed strain AKS211. All incubation steps during transformation and counter-selection were conducted at 37°C. Deletions were screened by PCR and validated by Sanger sequencing of PCR products from genomic DNA spanning the deletion (primers listed in Table 1). Whole genome Illumina sequencing was performed on phenol-chloroform-extracted genomic DNA (gDNA) to ensure the lack of second-site mutations. Because *Hbt. salinarum* is a polyploid organism, sequencing also verified the complete deletion of all copies of the *aglB* locus. The details of sequencing are as described in the ensuing paragraph.

**Table 1:** Primers used in this study.

Name	5′-3′ sequence	Purpose
Hs_aglB_up_F	CAGATCGAGCAGACGCATCTGGATC CACGAGACCAGCCAGTAGAACTCG	*aglB* deletion
Hs_aglB_up_R	GACGCCACGATCAGTGTCCCTCGCTC ATTGTGGAAACGG	*aglB* deletion
Hs_aglB_down_F	CCGTTTCCACAATGAGCGAGGGACAC TGATCGTGGCGTC	*aglB* deletion
Hs_aglB_down_R	GTATCTAGAACCGGTGACGTCACCAT GGGAGCAACACCATCGCACAGATC	*aglB* deletion
Hs_aglB_PCR_F	CCACGAGCTGTTGGAGGC	Sequencing
Hs_aglB_PCR_R	GGACTCACGACAGTCGTCG	Sequencing
Hs_aglB_seq_F	GACCAGCCAGTAGAACTCG	Sequencing
Hs_aglB_seq_R	GCAACACCATCGCACAGATC	Sequencing
pNBKO7_F	CAGATCGAGCAGACGCATCT	Sequencing
pNBKO7_R	GTATCTAGAACCGGTGACGT	Sequencing
FlaA1F	CAAGACCGCTAGTGGGACC	qPCR
FlaA1R	GCGTCGGCAGTGCTACC	qPCR
FlaA2F	ACCCTAACGCACGCCAAC	qPCR
FlaA2R	CGTTGTCGTTGTTCCCCTTG	qPCR
FlaB1F	CGAATCCATCAAGGGCAGC	qPCR
FlaB1R	GCTGCACCTCGTCACCAG	qPCR
FlaB2F	GAATTCGATTAAGGGCGACAAC	qPCR
FlaB2R	CAGTCCATTGGTGGTGATCT	qPCR
FlaB3F	CTCACGAAATCCACGATCCA	qPCR
FlaB3R	TGATGGATTCGGTGGTGAAG	qPCR
16SF	GGTACGTCTGGGGTAGGAGT	qPCR
16SR	AGACCCTAGCTTTCGTCCCT	qPCR

### Whole Genome Re-sequencing of the Δ*aglB* Strain

gDNA was extracted from 1 ml mid-logarithmic phase cultures of Δ*ura3* and Δ*aglB* using standard protocols. Briefly, pelleted cells were lysed in ddH_2_O and treated with RNaseI and Proteinase K. An equal volume of phenol-chloroform was added and the aqueous layer removed using Phase Lock Gel microfuge tubes (QuantaBio). DNA was ethanol precipitated and resuspended in modified TE buffer (10 mM Tris pH 8.0, 0.1 mM EDTA). DNA quality and concentration were measured using a Nanodrop spectrophotometer (Thermo Fisher Scientific). To shear, gDNA was diluted to 50 ng/μl in a 100 μl volume and sonicated in a Bioruptor Plus sonicating water bath (Diagenode) for 15–20 cycles, 30 s on, 90 s off, high setting. Resultant ragments (200–300 bp) were visualized by gel electrophoresis and ethidium bromide staining. DNA was submitted to the Duke Sequencing and Genomics Technologies core for Illumina TruSeq dual-index adapter ligation and library preparation. Samples were sequenced using an Illumina HiSeq 4000 (Sequencing and Genomics Technologies, Center for Genomic and Computational Biology, Duke University). 50 bp single read data were assessed for quality, aligned to *Hbt. salinarum* NRC-1 reference genome (RefSeq: NC_002607.1, NC_002608.1, NC_001869.1) (Ng et al., 2000), and analyzed using the *breseq* resequencing package using default parameters (Deatherage and Barrick, 2014). Raw sequencing data and the computational pipeline used in the *breseq* analysis can be accessed via the GitHub repository (see footnote 1). Strain AKS211 contained no reads within the *aglB* locus and no other detected single nucleotide polymorphisms (SNPs) or deletions at second sites relative to the Δ*ura3* parent strain. Raw sequencing data for both strains are available via the Sequence Read Archive at accession PRJNA526107.

### Enrichment of Archaellins

The five *Hbt. salinarum* archaellins (FlaA1, FlaA2, FlaB1, FlaB2, and FlaB3) were enriched from spent growth medium as previously described (Alam and Oesterhelt, 1984). Briefly, cultures were grown to logarithmic (OD_600_ ∼ 0.8) or stationary (OD_600_ ∼ 2.0) phase and held at room temperature without shaking for 24 h. The cultures were centrifuged for 30 min (6,000 × *g*, 15°C). The supernatant (post-spin 1 supernatant) was collected and centrifuged again for 15 min (16,000 × *g*, 15°C). The supernatant (post-spin 2 supernatant) was removed and the pelleted material was resuspended by shaking in 1 ml of 4 M basal salt solution (250 g NaCl, 20 g MgSO_4_⋅7H_2_O, 3 g sodium citrate, 2 g KCl per l) and heated for 10 min at 90°C. The heated suspension was centrifuged for 15 min (16,000 × *g*, 15°C). The resulting supernatant (post-spin 3 supernatant) was maintained at 4°C for 24 h and centrifuged for 2 h (40,000 × *g*, 4°C). After removal of the supernatant (post-spin 4 supernatant), the pellet (post-spin 4 pellet) was resuspended in sample buffer and separated by 12% SDS-PAGE and stained with Coomassie brilliant blue.

### Liquid Chromatography-Electrospray Ionization Mass Spectrometry (LC-ESI MS)

LC-ESI MS was conducted for identification and analysis of isolated *Hbt. salinarum* archaellins. Initially, in-gel digestion of bands containing these proteins was conducted. Gel bands containing the archaellins were excised using a clean scalpel, destained in 400 μl of 50% (vol/vol) acetonitrile (Sigma) in 40 mM NH_4_HCO_3_, pH 8.4, dehydrated with 100% acetonitrile, and dried using a SpeedVac drying apparatus. The proteins were reduced with 10 mM dithiothreitol (Sigma) in 40 mM NH_4_HCO_3_ at 56°C for 60 min and then alkylated for 45 min at room temperature with 55 mM iodoacetamide in 40 mM NH_4_HCO_3_. The gel pieces were washed with 40 mM NH_4_HCO_3_ for 15 min, dehydrated with 100% acetonitrile, and SpeedVac dried. The gel slices were rehydrated with 12.5 ng/μl of mass spectrometry (MS)-grade Trypsin (Pierce) in 40 mM NH_4_HCO_3_ and incubated overnight at 37°C. The protease-generated peptides were extracted with 0.1% (v/v) formic acid in 20 mM NH_4_HCO_3_, followed by sonication for 20 min at room temperature, dehydration with 50% (v/v) acetonitrile, and additional sonication. After three rounds of extraction, the gel pieces were dehydrated with 100% acetonitrile and dried completely with a SpeedVac. Next, 12.5 ng/μl Glu-C (V8) protease (Promega, sequencing-grade) in 40 mM NH_4_HCO_3_ was added. After an overnight incubation at 37°C, the sample was dried completely with a SpeedVac, resuspended in 5% (v/v) acetonitrile containing 1% formic acid (v/v) and infused into the mass spectrometer using static nanospray Econotips (New Objective, Woburn, MA). The protein digests were separated on-line by nano-flow reverse-phase liquid chromatography (LC) by loading onto a 150-mm by 75-μm (internal diameter) by 365-μm (external diameter) Jupiter pre-packed fused silica 5-μm C_18_ 300Å reverse-phase column (Thermo Fisher Scientific, Bremen, Germany). The sample was eluted into the LTQ Orbitrap XL mass spectrometer (Thermo Fisher Scientific) using a 60-min linear gradient of 0.1% formic acid (v/v) in acetonitrile/0.1% formic acid (1:19, by volume) to 0.1% formic acid in acetonitrile/0.1% formic acid (4:1, by volume) at a flow rate of 300 nl/min.

### Motility Assay

To assay motility, parent and Δ*aglB* stain cells were grown on semi-solid CM containing 0.3% agar (w/v). Aliquots (10 μl) of liquid cultures of parent or Δ*aglB* strain grown to logarithmic or stationary phase (OD_600_ ∼ 0.8 or 2.0, respectively) were placed at the center of the agar surface. The plates were incubated for 4 days at 42°C (Patenge et al., 2001), after which time the diameter of the motility halo was measured. Where the halos were not perfectly circular, the diameter was considered as the average of the longest and shortest linear spans of the halo area. Three plates each were assessed per strain type and growth phase. To confirm the viability of each strain after the 4 day-long period of incubation, cells from each plate were picked and grown for 4 days at 42°C in 10 ml of CM.

### Transmission Electron Microscopy

Cultures (2 ml) of parent and Δ*aglB* stain cells were pelleted (2 min at 8000 × *g* in a microfuge) and the supernatant was removed. The pellet was carefully resuspended in 1 ml basal salt solution (corresponding to *Hbt. salinarum* CM without peptone). The resulting cell suspension (1 ml) was pelleted as before, the supernatant was removed, and the pellet was resuspended in 1 ml of BSS.

Aliquots (2.5 μl) were applied to 300 mesh copper grid and any excess liquid was blotted with filter paper after 1 min. The grid was dried in air for 1 min, when 5 μl of uranyl acetate (2%) was applied for negative staining to increase the sample contrast. Next, the grids were blotted once more to remove excess uranyl acetate. Finally, the grids were dried in air before insertion into the microscope. Electron micrographs were taken with a FEI Tecnai T12 G^2^ TWIN transmission electron microscope operating at 120 kV.

### Quantitative Real-Time PCR (qRT-PCR)

To quantify the mRNA levels transcribed from archaellin genes, qRT-PCR was performed. Parent and Δ*aglB* strain cells were grown to logarithmic or stationary phase (OD_600_ ∼ 0.8 or 2.0, respectively). RNA was isolated from culture aliquots (1 ml) using an RNeasy mini-kit (Qiagen) according to the manufacturer’s instructions. Contaminating DNA in the RNA samples was eliminated with RNase-Free DNase Set (Qiagen) during RNA extraction. RNA concentration was determined spectrophotometrically using a Nanodrop. Single-stranded cDNA was prepared from the extracted RNA using random hexameric primers in a Superscript IV 1st Strand System (Invitrogen). Relative transcript levels were then determined by qPCR analysis using a CFX384TM Real Time System (Bio Rad). The reaction mix contained 5 μl of SYBR green mix (Applied Biosystems), 0.3 μM of primers (listed in Table 1), 5 ng cDNA and DDW in a total reaction volume of 10 μl. The following parameters were used: 95°C for 3 min, 40 cycles of 15 s at 95°C and 1 min at 60°C for annealing, extension and read fluorescence, respectively. Melting curve analysis was performed after each run to ensure the specificity of the products. The efficiency of each primer set was calculated using five-to-six serial dilutions of the wild type sample. Using this efficiency value for each primer set, relative expression was calculated using the standard 2^-ΔΔct^ formula (Pfaffl, 2001), with the *16S rRNA* gene as a reference. Statistical significance was determined using Student’s unpaired *t*-test to compare the level of each transcript in the parent versus mutant strains.

## Results

### *Hbt. salinarum* Archaellins Are *N*-Glycosylated by a Tetrasaccharide

**Figure 1:**
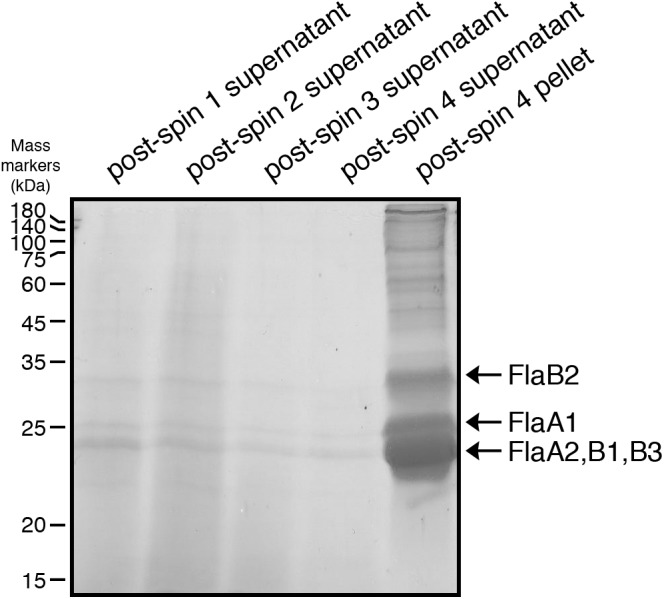
Enrichment of *Hbt. salinarum* archaellins. *Hbt. salinarum* archaellins were enriched from the growth medium of logarithmic phase cultures, as described in section “Materials and Methods.” Aliquots of the indicated fractions collected during isolation were separated by 12% SDS-PAGE and Coomassie-stained. The positions of molecular mass markers (in kDa) are indicated on the left, while the positions of the five *Hbt. salinarum* archaellins are indicated on the right.

**Figure 2:**
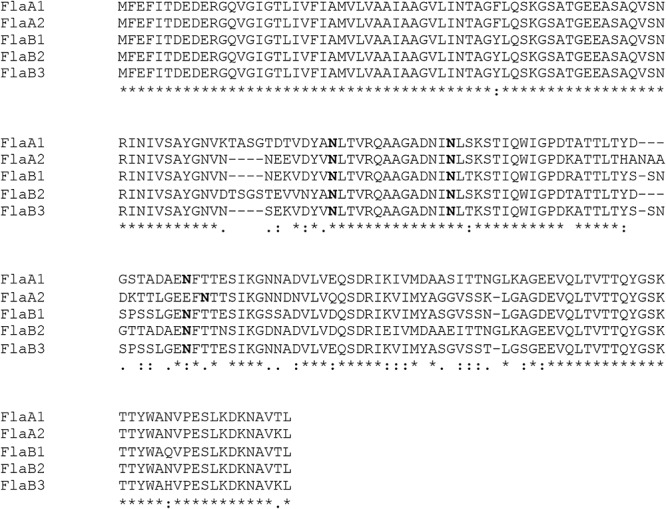
Alignment of the sequence of the five *Hbt. salinarum* archaellins. Sequence alignment was performed using ClustalW (https://npsa-prabi.ibcp.fr/cgi-bin/npsa_automat.pl?xpage=npsa_clustalw.html) with the default settings. In each sequence, potentially modified Asn residues are in bold. The line under the five sequences indicates the presence of identical (^∗^), highly similar (:) or similar (.) residues at each position.

*N*-glycosylation of *Hbt. salinarum* archaellins was first reported by Wieland et al. (1985), who described a sulfated tetrasaccharide comprising a glucose and three sulfated glucuronic acids or two glucoses and two sulfated glucuronic acids *N*-linked to the three archaellins then proposed to comprise the archaellum in this organism. To confirm these observations, archaellins were enriched from the spent growth medium of logarithmic and stationary phase cultures. SDS-PAGE of the enrichments revealed three archaellin-containing bands (Figure 1), as previously reported (Alam and Oesterhelt, 1984). Mass spectrometry of the Coomassie-stained bands identified the five *Hbt. salinarum* archaellins, namely the 31.5 kDa FlaB2 (VNG0961G), the 26.5 kDa FlaA1 (VNG1008G), and the 23.5 kDa FlaA2 (VNG1009G), FlaB1 (VNG0960G) and FlaB3 (VNG0962G) archaellins now known to exist (Gerl et al., 1989), although they could not be distinguished from each other on the Coomassie-stained gel. ClustalW alignment of the amino acid sequences of the precursor forms of these proteins showed their considerable shared identity (Figure 2). For instance, all five archaellins contain three putative sites of *N*-glycosylation found at identical or almost identical positions. To determine which of these sites are indeed modified, proteolytic fragments generated from the five archaellins were analyzed by LC-ESI MS to identify Asn-bound glycans.

**Figure 3:**
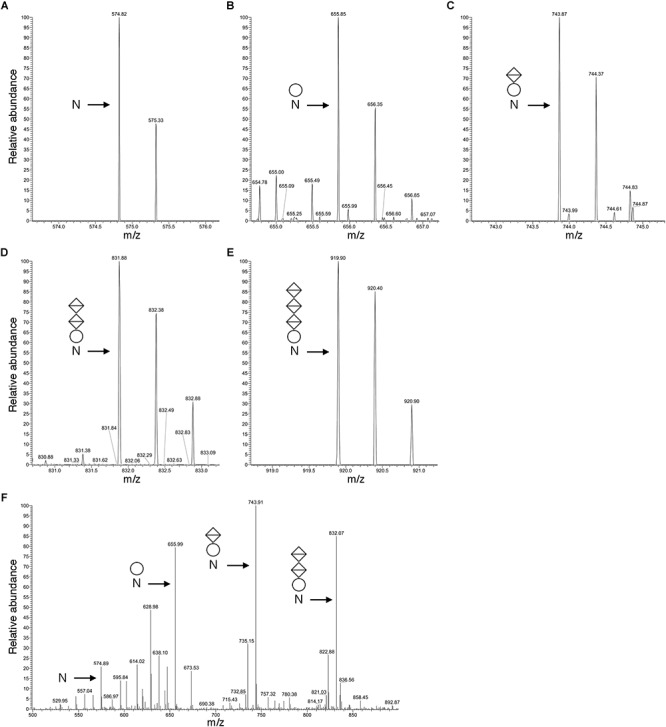
Archaellin *N*-glycosylation revealed by LC-ESI MS. **(A–E)** As an example of archaellin *N*-glycosylation, the glycosylation profile of the QAAGADNINLSK peptide generated from FlaA1, FlaA2 and FlaB2 is presented. The monoisotopic [M+2H]^2+^ ion peaks of **(A)** the non-modified peptide and **(B)** the same peptide modified by a hexose, **(C)** by a hexose and a hexuronic acid, **(D)** by a hexose and two hexuronic acids, and **(E)** by a hexose and three hexuronic acids are shown. **(F)** MS/MS analysis of the tetrasaccharide-charged peptide reveals a fragmentation pattern consistent with the peptide modified by a hexose and a hexose and 1-3 hexuronic acids, as well as the non-modified peptide. In each panel, N corresponds to the modified Asn residue, the circle corresponds to a hexose and the diamonds with a horizontal bar correspond to hexuronic acids.

Figure 3 presents a representative *N*-glycosylation profile of one of these peptides, namely the sequence QAAGADNINLSK common to FlaA1, FlaA2 and FlaB2, generated following digestion with trypsin and Glu-C protease. Such analysis revealed a peak of *m/z* 574.82 (Figure 3A), corresponding to the [M+2H]^2+^ ion of the peptide (calculated mass *m/z* 574.82), containing a single putative *N*-glycosylation site (Asn-97, Asn-69 and Asn-73 in FlaA1, FlaA2 and FlaB2, respectively). Peaks of *m/z* 655.85, 743.87, 831.88 and 919.90 (Figure 3B–E) were also detected, consistent with calculated masses of the same Asn-containing peptide modified by a hexose (calculated mass *m/z* 655.82; Figure 3B), a hexose and a hexuronic acid (calculated mass *m/z* 743.82; Figure 3C), a hexose and two hexuronic acids (*m/z* 831.82; Figure 3D), and a hexose and three hexuronic acids (*m/z* 919.82; Figure 3E), respectively. MS/MS analysis of the [M+2H]^2+^ ion of the peptide at *m/z* 919.93 yielded a fragmentation pattern consistent with modification by the precursors of a tetrasaccharide comprising a hexose and three hexuronic acids. Specifically, peaks corresponding to the non-modified peptide (*m/z* 574.89), as well as the same peptide modified by a hexose (*m/z* 655.99), a hexose and a hexuronic acid (*m/z* 743.91), and a hexose and two hexuronic acids (*m/z* 832.07) were seen (Figure 3F). Similar *N*-glycosylation profiles were also seen with other archaellin-derived peptides (Table 2), including the FlaB1- and FlaB3-derived QAAGADNINLTK peptide first observed in the original report of *Hbt. salinarum* archaellin *N*-glycosylation (Wieland et al., 1985). The MS/MS profiles of these other archaellin-derived peptides modified by a hexose and three hexuronic acids are presented in Supplementary Figure S1. At the same time, no evidence for sulfated hexuronic acids was obtained, nor was there any indication of modification by an *N*-linked tetrasaccharide comprising two hexoses and two hexuronic acids, as reported previously (Wieland et al., 1985).

**Table 2:** Glycosylated Asn residues in *Hbt. salinarum* archaellins.

Archaellin	Sequence^1^	Observed mass (*m/z*)^2^	Calculated mass (*m/z*)^2^	Bound glycan
FlaA1	TASGTDTVDYA^84^NLTVR	842.42	842.41	
		923.44	923.41	Hex
		1011.46	1011.41	Hex, HexA
		1099.48	1099.41	Hex, HexA_2_
		1187.49	1187.41	Hex, HexA_3_
FlaA1/FlaA2/FlaB2^3^	QAAGADNI^97/69/73^NLSK	601.31	601.31	
		682.34	682.31	Hex
		770.35	770.31	Hex, HexA
		858.37	858.31	Hex, HexA_2_
		946.39	946.31	Hex, HexA_3_
FlaA2	F^102^NTTSIK	n.d.^4^	405.72	
		n.d.	486.72	Hex
		574.79	574.72	Hex, HexA
		662.80	662.72	Hex, HexA_2_
		750.79	750.72	Hex, HexA_3_
FlaA2/FlaB1/FlaB3^3^	VDYV^56/80/56^NLTVR	539.80	539.80	
		620.82	620.80	Hex
		708.84	708.80	Hex, HexA
		796.85	796.80	Hex, HexA_2_
		884.87	884.80	Hex, HexA_3_
FlaB1/FlaB3^3^	QAAGADNI^93/69^NLTK^5^	608.31	608.32	
		689.35	689.32	Hex
		777.36	777.32	Hex, HexA
		865.38	865.32	Hex, HexA_2_
		953.39	953.32	Hex, HexA_3_
FlaB2	VVNYA^60^NLTVR	574.82	574.82	
		655.85	655.82	Hex
		743.87	743.82	Hex, HexA
		831.88	831.82	Hex, HexA_2_
		919.90	919.82	Hex, HexA_3_

^1^*The modified Asn in the sequence is numbered. ^*2*^All values correspond to the mass (*m/z*) of the [M+2H]^*2*+^ species; ^*3*^The same peptide is generated from the archaellins listed. The position of the modified Asn in each archaellin is provided; ^*4*^n.d. – not detected; and ^*5*^Glycosylation of the same peptide was reported by Wieland et al. (1985)*.

### *Hbt. salinarum* Cells Deleted of *aglB* Are Impaired for Flotation and Motility

**Figure 4:**
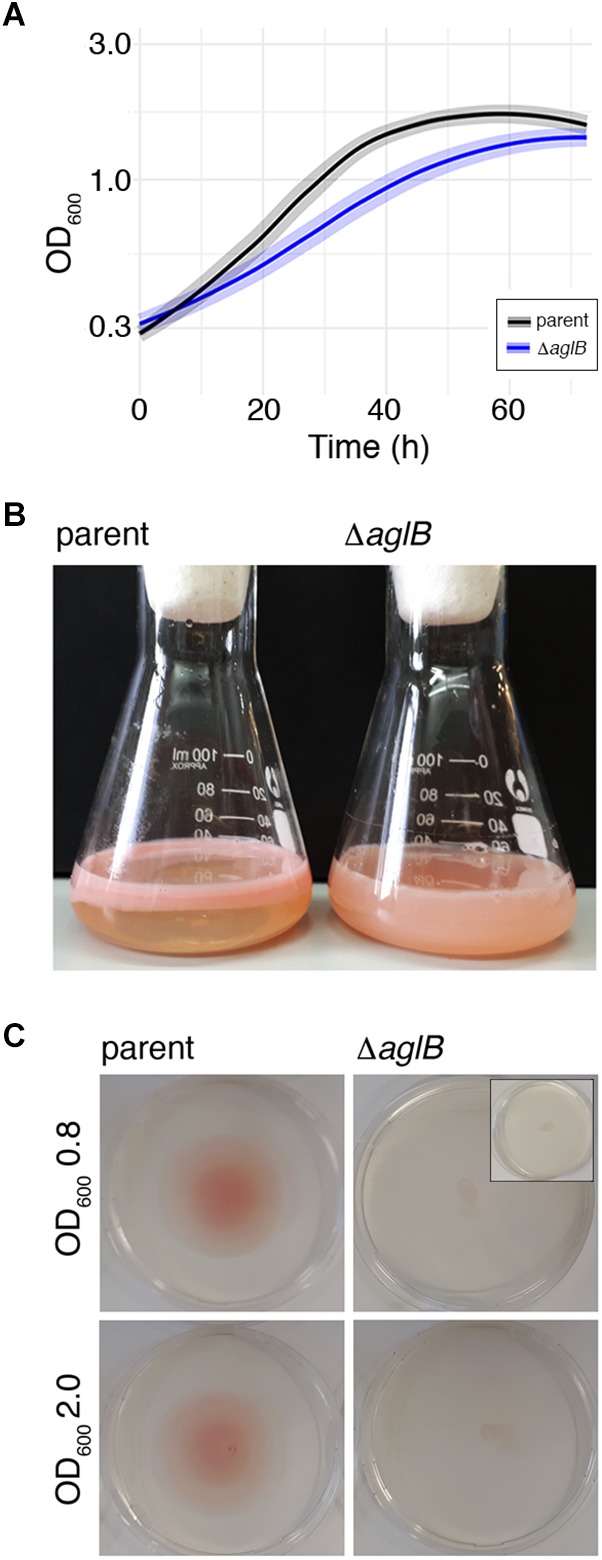
*Halobacterium salinarum* Δ*aglB* cells show modified growth and compromised flotation and motility. **(A)** Growth curves of *Hbt. salinarum Δura3* parent (dark gray curve) and *ΔaglB* mutant (blue curve) cultures. Dark lines represent loess-smoothed average data of five biological replicate cultures and three technical replicates of each. Shaded regions represent standard deviation from the mean. **(B)** *Hbt. salinarum* parent (left) and Δ*aglB* (right) strain cultures were grown to stationary phase and left standing at room temperature. **(C)** Representative plates upon which aliquots (10 μl) of parent or Δ*aglB* strain liquid cultures grown to logarithmic or stationary phase (OD_600_ ∼ 0.8 or 2.0, respectively) were spotted at the plate center. Motility halos were seen on plates containing parent but not deletion strain cells after a four-day incubation at 42°C. The inset in the upper right panel shows the amount of culture originally plated.

To assess the importance of archaellin *N*-glycosylation in *Hbt. salinarum*, a strain deleted of *VNG1068G* (*aglB*), encoding the oligosaccharyltransferase, was constructed. *Hbt. salinarum* AglB shares 47% identity with *Hfx. volcanii* AglB, known to be required for *N*-glycosylation in this species (Abu-Qarn et al., 2007), and was able to functionally replace its *Hfx. volcanii* counterpart (Cohen-Rosenzweig et al., 2014). Whole genome re-sequencing of the Δ*aglB* strain demonstrated that all copies of the *aglB* gene were deleted from this polyploid organism and that second site suppressor mutations were absent (Supplementary Table S1, see also GitHub footnote 1). As reported for other euryarchaeal species, such as *Hfx. volcanii* (Abu-Qarn and Eichler, 2006), *M. voltae* (Chaban et al., 2006) and *M. maripaludis* (VanDyke et al., 2009), the viability of the *Hbt. salinarum* Δ*aglB* strain points to the fact that *N*-glycosylation is not essential in this organism. The Δ*aglB* strain did, however, exhibit a slight but significant growth defect during logarithmic phase, relative to the *Hbt. salinarum* parent strain under standard growth conditions (1.8-fold lower growth rate during logarithmic phase; Welch’s two-sample *t*-test, *p* < 3.77 × 10^-7^; Figure 4A and Supplementary Table S2).

When left standing after reaching stationary phase, differences in the appearance of the parent and deletion strain cultures were apparent. Whereas cells in the parent strain culture had migrated toward the surface of the growth medium, preferentially near the glass-medium interface in the Erlenmeyer vessel used to grow the cells (Figure 4B, left), cells of the deletion stain remained dispersed throughout the growth medium (Figure 4B, right). Since qRT-PCR showed no differences in the levels of gas vesicle-related *gvpA* transcripts, encoding the major gas vesicle structural protein (Pfeifer, 2015), it would appear that gas vesicle assembly and/or function were not affected by the absence of AglB (parent strain: 1.0 ± 0.07 (standard error), *n* = 3; Δ*aglB* strain: 1.06 ± 0.28, *n* = 3). To confirm that the failure of mutant cells to reach the medium surface instead involved perturbed motility, cell migration on soft agar plates was assayed. When parent strain cells grown to mid-logarithmic phase (OD_600_ ∼ 0.8) were applied to 0.3% agar-containing plates, a circular zone 4.9 ± 0.17 cm in diameter (*n* = 3) was observed (Figure 4C, upper left panel). In contrast, Δ*aglB* cells grown to a similar OD migrated to a zone only 0.93 ± 0.1 cm in diameter (*n* = 3; Figure 4B, upper right panel). When the same experiment was repeated using parent and Δ*aglB* strain cultures grown to stationary phase (OD_600_ ∼ 2.0), circular zones with diameters of 4.6 ± 0.4 cm (*n* = 3) and 1.1 ± 0.06 cm (*n* = 3) were measured (Figure 4B, lower left and right panels, respectively). Since the area covered by the Δ*aglB* strain at the start of the experiment was similar to that covered by the applied 10 μl aliquot originally applied to the plates (Figure 4C, inset in upper right panel), it can be concluded that the mutant cells are non-motile or only weakly motile. To confirm that both the plated parent and Δ*aglB* strains had remained viable throughout the assay, cells on the plates were transferred to liquid medium. Both cultures reached saturation (OD_600_ ∼ 2.0) after 4 days of growth, confirming that the mutant strain cells were viable throughout the assay. Taken together, this phenotypic characterization suggests that AglB, and hence *N*-glycosylation, plays a role in normal cell growth, flotation and migration.

### *Hbt. salinarum* Δ*aglB* Cells Lack Archaella

**Figure 5:**
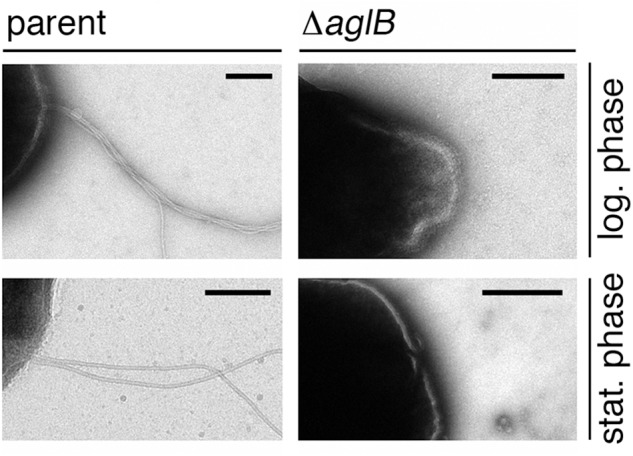
*Halobacterium salinarum* Δ*aglB* cells do not present archaella. Parent (left panels) or Δ*aglB* (right panels) strain cultures grown to logarithmic (log.; upper panels) or stationary (stat.; lower panels) phase (OD_600_ ∼ 0.8 or 2.0, respectively) to determine whether or not archaella were present. The scale bars in each panel corresponds to 0.2 μm, except that in the lower right panel, which corresponds to 0.5 μm.

To directly assess whether the compromised flotation and motility seen in the deletion strain cells reflected decreased archaellin levels or archaellum assembly, cells of the parent and Δ*aglB* strains grown to either logarithmic or stationary phase were examined by transmission electron microscopy. At both stages of growth, archaella were readily detected in the parent strain (Figure 5, right panels). In contrast, no archaella attached to the deletion strain cells could be detected at either growth stage (Figure 5, left panels). As such, it appears the *N*-glycosylation is necessary for *Hbt. salinarum* archaellum assembly and/or cellular attachment.

To distinguish between these two possibilities, the same protocol used to enrich for archaellin proteins from parent strain cells was employed with Δ*aglB* strain cells. Whereas the archaellins were easily detected and isolated from the medium of stationary phase parent strain cells (Figure 1), barely detectable quantities were obtained from an equivalent amount of growth medium from mutant strain cells grown to the same density, as revealed by SDS-PAGE and Coomassie staining (Supplementary Figure S2). Mass spectrometry, providing higher sensitivity, confirmed that the medium of the mutant cells indeed contained archaellins, yet also showed that these were not *N*-glycosylated (Supplementary Figure S3).

### Deletion of *Hbt. salinarum aglB* Affects Archaellin Transcript Levels

**Figure 6:**
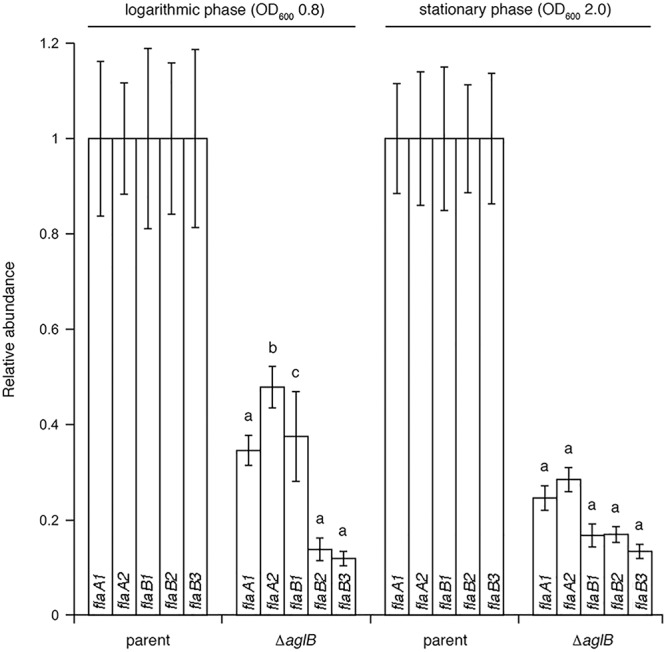
qRT-PCR reveals reduced transcription of archaellin-encoding mRNA in *Halobacterium salinarum* Δ*aglB* cells. Transcript levels isolated from parent or Δ*aglB* strain cells grown to logarithmic or stationary phase (OD_600_ ∼ 0.8 or 2.0, respectively) were quantified. The relative abundance of the different archaellin transcripts are normalized to the value calculated for the parent strain at the same growth phase. The values recorded at logarithmic phase represent the average of three biological repeats, each comprising eight technical repeats. The values recorded at stationary phase represent the average of three biological repeats, each comprising four technical repeats. Statistical significance is denoted as follows: a, *p* < 0.0001; b, *p* < 0.0005; c, *p* < 0.01). Error bars represent ± SEM.

To determine whether the substantially diminished amount of archaellins found in the growth medium of the deletion strain was the result of reduced transcription of archaellin-encoding genes, qRT-PCR was performed to compare *flaA1*, *flaA2*, *flaB1*, *flaB2* and *flaB3* transcript levels in the parent and Δ*aglB* strains. Significantly higher levels of *flaA1*, *flaA2*, *flaB1*, *flaB2* and *flaB3* transcripts were detected in parent strain cultures, relative to mutant strain cultures at the same growth stage (Figure 6). When the levels of mRNA for each archaellin in parent and mutant strain cultures were compared as a function of growth phase, higher *flaA1*, *flaA2*, *flaB1*, *flaB2* and *flaB3* transcript levels were detected in logarithmic phase than in stationary phase cultures in both strains (Supplementary Figure S4). Taken together, these data show that transcript levels encoding archaellins are lower in the *aglB* deletion stain, which would explain the absence of archaella and the observed decrease in archaellin protein levels released into the growth medium.

## Discussion

Reports that appeared over three decades ago, when archaeal *N*-glycosylation was first reported in *Hbt. salinarum*, provided important biochemical insight into this process (Lechner et al., 1985a,b; Wieland et al., 1985; Paul et al., 1986). More recently, components involved in the *Hbt. salinarum N*-glycosylation pathway have been defined (Cohen-Rosenzweig et al., 2014; Kandiba and Eichler, 2015). Still, the significance of such post-translational modification in *Hbt. salinarum*, and indeed across Archaea, largely remains an open question (Koomey and Eichler, 2017). In the present report, insight into the importance of *N*-glycosylation in *Hbt. salinarum*, and specifically the *N*-glycosylation of archaellins, was provided.

The present study provided the first direct demonstration that *Hbt. salinarum* AglB is necessary for *N*-glycosylation. Previous efforts had shown that *Hbt. salinarum* AglB could functionally replace its *Hfx. volcanii* counterpart, where the *N*-linked glycan that decorates cell surface glycoproteins is also assembled on a dolichol phosphate carrier (Guan et al., 2010; Cohen-Rosenzweig et al., 2014). It is still not clear, however, whether or not *Hbt. salinarum* AglB also processes the distinct glycan assembled on dolichol pyrophosphate carriers and transferred to the Asn-2 position of the S-layer glycoprotein (Mescher and Strominger, 1978; Paul et al., 1986; Cohen-Rosenzweig et al., 2014). Accordingly, the deletion of *Hfx. volcanii aglB* only affected the attachment of one of the two distinct *N*-linked glycans that can decorate the S-layer glycoprotein, suggesting the existence of a novel archaeal oligosaccharyltransferase (Kaminski et al., 2013). The present report also demonstrated the important physiological role of archaellin *N*-glycosylation in *Hbt. salinarum*. While not essential for survival, *N*-glycosylation is nonetheless needed for wild type cell growth, flotation and motility, and archaellum assembly. In the absence of AglB, and hence archaellin *N*-glycosylation, *Hbt. salinarum* cells were neither able to reach the medium surface when grown in liquid culture, nor were they able to spread on agar swim plates. These findings agree with the results of earlier efforts showing that in the absence of AglB, motility and archaellum assembly were lost in *M. voltae*, *M. maripaludis* and *Hfx. volcanii* (Chaban et al., 2006; VanDyke et al., 2009; Tripepi et al., 2012). However, archaellin levels in the *M. maripaludis* and *Hfx. volcanii* mutants and the respective parent strains remained similar (VanDyke et al., 2009; Tripepi et al., 2012). In contrast, it was shown here that *Hbt. salinarum* Δ*aglB* cells do not assemble archaella and released far lower levels of archaellins into the growth medium than did the parent strain. As the level of a gas vesicle-related transcript was equivalent between the *aglB* deletion and parent strains, it is unlikely that compromised assembly and/or function of these entities, which serve as flotation devices in *Hbt. salinarum* (Pfeifer, 2015), contribute to the perturbed distribution and movement of the mutant strain in the flotation and swarming assays, respectively. Instead, it is likely that perturbed archaellin levels and subsequently, archaellum assembly, are responsible.

In assessing the composition of the *N*-linked glycan decorating *Hbt. salinarum* archaellins, mass spectrometry revealed it to correspond to a tetrasaccharide comprising a hexose and three hexuronic acids, consistent with earlier studies (Wieland et al., 1985). At that time, the same tetrasaccharide was reported to be assembled on a dolichol phosphate carrier and also *N*-linked to the S-layer glycoprotein in this haloarchaeon (Wieland et al., 1983; Lechner et al., 1985a,b). However, in contrast to previous studies, no mass spectrometry evidence for the sulfation of these sugars was obtained here. Indeed, a later study detected dolichol phosphate modified by a hexose and a hexuronic acid in a total extract of *Hbt. salinarum* lipids that presumably serves as a precursor of the *N*-linked tetrasaccharide, yet not the sulfated version of the latter sugar in the disaccharide-modified lipid carrier (Cohen-Rosenzweig et al., 2014). In addition, the cluster of *Hbt. salinarum* genes assigned roles in the biogenesis of the *N*-linked tetrasaccharide does not include any sequence encoding a sulfotransferase (Kandiba and Eichler, 2015). As it is unlikely that sulfate groups bound to *N*-linked glycan sugars were lost during the preparation of archaellins for mass spectrometry in the present study [or the dolichol phosphate-bound precursor detected in a previous effort (Cohen-Rosenzweig et al., 2014)], the source of the discrepancy is not clear. In the earlier studies, glucuronic acid sulfation at the lipid carrier and glycoprotein levels was demonstrated by *in vivo* radiolabeling with carrier-free ^35^SO_4_^2-^ (Wieland et al., 1980, 1985; Lechner et al., 1985a). It is thus conceivable that, at both the dolichol phosphate- and the protein-bound levels, the tetrasaccharide contains only very minor levels of sulfation which could be visualized using a radiolabel yet that was not detected here by mass spectrometry. At the same time, the presence or absence of *N*-linked tetrasaccharide sulfation could reflect environmental considerations, given recent reports linking modified *N*-glycosylation to growth conditions (Kaminski et al., 2013; Ding et al., 2016).

The reduced level of archaellins detected in the spent growth medium of the mutant strain likely reflects reduced *fla* transcript levels. Presently, our understanding of archaellin gene expression regulation is only partial and limited to a few species. Whereas starvation (i.e., growth in the absence of tryptone) was shown to induce archaellation in *S. acidocaldarius* and *Sulfolobus solfataricus* (Szabó et al., 2007; Lassak et al., 2012), elsewhere archaellation is influenced by environmental conditions. For example, in *M. maripaludis*, temperature affects archaellum expression (Ding et al., 2016), while in *Haloarcula marismortui*, environmental salinity impacts archaellin expression patterns (Syutkin et al., 2019). In the case of *Hfx. volcanii*, a conserved region of pili involved in adhesion regulates archaellin biosynthesis (Esquivel and Pohlschröder, 2014). Post-translational regulation also appears to be at play in *Hbt. salinarum*, as reported here, with a protein-processing event, namely *N*-glycosylation, seemingly regulating the appearance of archaella. Although the mode by which *N*-glycosylation regulates archaellin transcription remains to be defined, transcriptional regulation of metabolic enzyme-coding genes has been reported to have an indirect effect on protein glycosylation in *Hbt. salinarum* (Todor et al., 2014).

Finally, the apparent relation between *N*-glycosylation and archaellin gene transcription described here agrees with the findings of a recent proteomics study, which reported a major decrease in the levels of normally *N*-glycosylated proteins in *Campylobacter jejuni* cells lacking the bacterial oligosaccharyltransferase PglB, together with an increase in the level of stress-related proteins (Cain et al., 2019). Although it remains to be determined whether deletion of *aglB* has a similar global effect in *Hbt. salinarum*, the findings reported here may reflect a novel role for archaeal *N*-glycosylation in glycoprotein gene expression. Given the role of the *Hbt. salinarum* archaellum in photo-, aero- and chemotaxis (Marwan et al., 1991), the impact of arrested *N*-glycosylation, replicated here via *aglB* deletion, on archaellin levels and archaellum assembly, could reflect a program relevant to the natural environment.

Taken together, the genetic, phenotyping, and mass spectrometry evidence provided in the present report reveals that *Hbt. salinarum* AglB is required for *N*-glycosylation of archaellin proteins and archaellum assembly, and that this post-translational modification is important for cell growth and motility, as well as archaellin gene expression.

## Data Availability

The whole genome resequencing datasets generated for this study can be found in the Sequence Read Archive (accession PRJNA526107), with data analysis and code available via the GitHub repository (https://github.com/amyschmid/aglB-WGS-growth). Raw growth curve data and analysis code are also available via the GitHub repository.

## Author Contributions

MZ and CD performed the experiments. All authors analyzed the data. JE wrote the manuscript with contributions from all authors.

## Conflict of Interest Statement

The authors declare that the research was conducted in the absence of any commercial or financial relationships that could be construed as a potential conflict of interest.

## Footnotes

1 https://github.com/amyschmid/aglB-WGS-growth

## References

[B1] Abu-Qarn M. Eichler J. (2006). Protein N-glycosylation in Archaea: defining *Haloferax volcanii* genes involved in S-layer glycoprotein glycosylation. *Mol. Microbiol.* 61 511–525. 10.1111/j.1365-2958.2006.05252.x 16762024

[B2] Abu-Qarn M. Yurist-Doutsch S. Giordano A. Trauner A. Morris H. R. Hitchen P. (2007). Haloferax volcanii AglB and AglD are involved in N-glycosylation of the S-layer glycoprotein and proper assembly of the surface layer. *J. Mol. Biol.* 374 1224–1236. 10.1016/j.jmb.2007.10.042 17996897

[B3] Alam M. Oesterhelt D. (1984). Morphology, function and isolation of halobacterial flagella. *J. Mol. Biol.* 176 459–475. 10.1016/0022-2836(84)90172-4 6748081

[B4] Cain J. A. Dale A. L. Niewold P. Klare W. P. Man L. White M. Y. (2019). Proteomics reveals multiple phenotypes associated with N-linked glycosylation in *Campylobacter jejuni*. *Mol. Cell Proteomics* 18 715–734. 10.1074/mcp.RA118.001199 30617158 PMC6442361

[B5] Chaban B. Logan S. M. Kelly J. F. Jarrell K. F. (2009). AglC and AglK are involved in biosynthesis and attachment of diacetylated glucuronic acid to the N-glycan in *Methanococcus voltae*. *J. Bacteriol.* 191 187–195. 10.1128/JB.00885-08 18978056 PMC2612417

[B6] Chaban B. Voisin S. Kelly J. Logan S. M. Jarrell K. F. (2006). Identification of genes involved in the biosynthesis and attachment of *Methanococcus voltae* N-linked glycans: insight into N-linked glycosylation pathways in Archaea. *Mol. Microbiol.* 61 259–268. 10.1111/j.1365-2958.2006.05226.x 16824110

[B7] Cohen-Rosenzweig C. Guan Z. Shaanan B. Eichler J. (2014). Substrate promiscuity: AglB, the archaeal oligosaccharyltransferase, can process a variety of lipid-linked glycans. *Appl. Environ. Microbiol.* 80 486–496. 10.1128/AEM.03191-13 24212570 PMC3911113

[B8] Darnell C. L. Tonner P. D. Gulli J. G. Schmidler S. C. Schmid A. K. (2017). Systematic discovery of archaeal transcription factor functions in regulatory networks through quantitative phenotyping analysis. *mSystems* 2 e32–e17. 10.1128/mSystems.00032-17 28951888 PMC5605881

[B9] Deatherage D. E. Barrick J. E. (2014). Identification of mutations in laboratory-evolved microbes from next-generation sequencing data using breseq. *Methods Mol. Biol.* 1151 165–188. 10.1007/978-1-4939-0554-6_12 24838886 PMC4239701

[B10] Ding Y. Jones G. M. Uchida K. Aizawa S. I. Robotham A. Logan S. M. (2013). Identification of genes involved in the biosynthesis of the third and fourth sugars of the *Methanococcus maripaludis* archaellin N-linked tetrasaccharide. *J. Bacteriol.* 195 4094–4104. 10.1128/JB.00668-13 23836872 PMC3754732

[B11] Ding Y. Lau Z. Logan S. M. Kelly J. F. Berezuk A. Khursigara C. M. (2016). Effects of growth conditions on archaellation and N-glycosylation in *Methanococcus maripaludis*. *Microbiology* 162 339–350. 10.1099/mic.0.000221 26643118

[B12] Ding Y. Uchida K. Aizawa S. Murphy K. Berezuk A. Khursigara C. M. (2015). Effects of N-glycosylation site removal in archaellins on the assembly and function of archaella in *Methanococcus maripaludis*. *PLoS One* 10:e0116402. 10.1371/journal.pone.0116402 25700084 PMC4336324

[B13] Esquivel R. N. Pohlschröder M. (2014). A conserved type IV pilin signal peptide H-domain is critical for the post-translational regulation of flagella-dependent motility. *Mol. Microbiol.* 93 494–504. 10.1111/mmi.12673 24945931

[B14] Gerl L. Deutzmann R. Sumper M. (1989). Halobacterial flagellins are encoded by a multigene family. identification of all five gene products. *FEBS Lett.* 244 137–140. 10.1016/0014-5793(89)81179-2 2924901

[B15] Gibson D. G. Young L. Chuang R. Y. Venter J. C. Hutchison C. A. III Smith H. O. (2009). Enzymatic assembly of DNA molecules up to several hundred kilobases. *Nat. Methods* 6 343–345. 10.1038/nmeth.1318 19363495

[B16] Guan Z. Naparstek S. Kaminski L. Konrad Z. Eichler J. (2010). Distinct glycan-charged phosphodolichol carriers are required for the assembly of the pentasaccharide N-linked to the *Haloferax volcanii* S-layer glycoprotein. *Mol. Microbiol.* 78 1294–1303. 10.1111/j.1365-2958.2010.07405.x 21091511 PMC3074503

[B17] Jarrell K. F. Albers S. V. (2012). The archaellum: an old motility structure with a new name. *Trends Microbiol.* 20 307–312. 10.1016/j.tim.2012.04.007 22613456

[B18] Jarrell K. F. Ding Y. Meyer B. H. Albers S. V. Kaminski L. Eichler J. (2014). N-linked glycosylation in Archaea: a structural, functional, and genetic analysis. *Microbiol. Mol. Biol. Rev.* 78 304–341. 10.1128/MMBR.00052-13 24847024 PMC4054257

[B19] Jarrell K. F. Jones G. M. Kandiba L. Nair D. B. Eichler J. (2010). S-layer glycoproteins and flagellins: reporters of archaeal posttranslational modifications. *Archaea* 2010:612948. 10.1155/2010/612948 20721273 PMC2913515

[B20] Jones G. M. Wu J. Ding Y. Uchida K. Aizawa S. Robotham A. (2012). Identification of genes involved in the acetamidino group modification of the flagellin N-linked glycan of *Methanococcus maripaludis*. *J. Bacteriol.* 194 2693–2702. 10.1128/JB.06686-11 22408155 PMC3347211

[B21] Kaminski L. Guan Z. Yurist-Doutsch S. Eichler J. (2013). Two distinct N-glycosylation pathways process the *Haloferax volcanii* S-layer glycoprotein upon changes in environmental salinity. *MBio* 4 e716–e713. 10.1128/mBio.00716-13 24194539 PMC3892788

[B22] Kandiba L. Eichler J. (2015). Deciphering a pathway of *Halobacterium salinarum* N-glycosylation. *MicrobiologyOpen* 4 28–40. 10.1002/mbo3.215 25461760 PMC4335974

[B23] Kelly J. Logan S. M. Jarrell K. F. VanDyke D. J. Vinogradov E. (2009). A novel N-linked flagellar glycan from *Methanococcus maripaludis*. *Carbohydr. Res.* 344 648–653. 10.1016/j.carres.2009.01.006 19203750

[B24] Koomey J. M. Eichler J. (2017). Sweet new roles for protein glycosylation in prokaryotes. *Trends Microbiol.* 25 662–672. 10.1016/j.tim.2017.03.001 28341406

[B25] Lassak K. Neiner T. Ghosh A. Klingl A. Wirth R. Albers S. V. (2012). Molecular analysis of the crenarchaeal flagellum. *Mol. Microbiol.* 83 110–124. 10.1111/j.1365-2958.2011.07916.x 22081969

[B26] Lechner J. Wieland F. (1989). Structure and biosynthesis of prokaryotic glycoproteins. *Annu. Rev. Biochem.* 58 173–194. 10.1146/annurev.biochem.58.1.173 2673008

[B27] Lechner J. Wieland F. Sumper M. (1985a). Biosynthesis of sulfated saccharides N- glycosidically linked to the protein via glucose. purification and identification of sulfated dolichyl monophosphoryl tetrasaccharides from halobacteria. *J. Biol. Chem.* 260 860–866. 2857171

[B28] Lechner J. Wieland F. Sumper M. (1985b). Transient methylation of dolichyl oligosaccharides is an obligatory step in halobacterial sulfated glycoprotein biosynthesis. *J. Biol. Chem.* 260 8984–8989. 4019460

[B29] Marwan W. Alam M. Oesterhelt D. (1991). Rotation and switching of the flagellar motor assembly in *Halobacterium halobium*. *J. Bacteriol.* 173 1971–1977. 10.1128/jb.173.6.1971-1977.1991 2002000 PMC207729

[B30] Mescher M. F. Strominger J. L. (1976). Purification and characterization of a prokaryotic glucoprotein from the cell envelope of *Halobacterium salinarum*. *J. Biol. Chem.* 251 2005–2014. 1270419

[B31] Mescher M. F. Strominger J. L. (1978). Glycosylation of the surface glycoprotein of *Halobacterium salinarum* via a cyclic pathway of lipid-linked intermediates. *FEBS Lett.* 89 37–41. 10.1016/0014-5793(78)80517-1 658399

[B32] Meyer B. H. Albers S. V. (2014). AglB, catalyzing the oligosaccharyl transferase step of the archaeal N-glycosylation process, is essential in the thermoacidophilic crenarchaeon *Sulfolobus acidocaldarius*. *MicrobiologyOpen* 3 531–543. 10.1002/mbo3.185 24916761 PMC4287180

[B33] Meyer B. H. Birich A. Albers S. V. (2015). N-Glycosylation of the archaellum filament is not important for archaella assembly and motility, although N-glycosylation is essential for motility in *Sulfolobus acidocaldarius*. *Biochimie* 118 294–301. 10.1016/j.biochi.2014.10.018 25447136

[B34] Ng W. V. Kennedy S. P. Mahairas G. G. Berquist B. Pan M. Shukla H. D. (2000). Genome sequence of *Halobacterium* species NRC-1. *Proc. Natl. Acad. Sci. U.S.A.* 97 12176–12181. 11016950 10.1073/pnas.190337797 PMC17314

[B35] Patenge N. Berendes A. Engelhardt H. Schuster S. C. Oesterhelt D. (2001). The fla gene cluster is involved in the biogenesis of flagella in *Halobacterium salinarum*. *Mol. Microbiol.* 41 653–663. 10.1046/j.1365-2958.2001.02542.x 11532133

[B36] Paul G. Lottspeich F. Wieland F. (1986). Asparaginyl-N-acetylgalactosamine: linkage unit of halobacterial glycosaminoglycan. *J. Biol. Chem.* 261 1020–1024. 3944078

[B37] Peck R. F. DasSarma S. Krebs M. P. (2000). Homologous gene knockout in the archaeon *Halobacterium salinarum* with ura3 as a counterselectable marker. *Mol. Microbiol.* 35 667–676. 10.1046/j.1365-2958.2000.01739.x 10672188

[B38] Pfaffl M. W. (2001). A new mathematical model for relative quantification in real-time RT-PCR. *Nucleic Acids Res.* 29:e45. 10.1093/nar/29.9.e45 PMC55695 11328886

[B39] Pfeifer F. (2015). Haloarchaea and the formation of gas vesicles. *Life* 5 385–402. 10.3390/life5010385 25648404 PMC4390858

[B40] Sharma K. Gillum N. Boyd J. L. Schmid A. K. (2012). The RosR transcription factor is required for gene expression dynamics in response to extreme oxidative stress in a hypersaline-adapted archaeon. *BMC Genomics* 13:351. 10.1186/1471-2164-13-351 22846541 PMC3443676

[B41] Siu S. Robotham A. Logan S. M. Kelly J. F. Uchida K. Aizawa S. (2015). Evidence that biosynthesis of the second and third sugars of the archaellin tetrasaccharide in the archaeon *Methanococcus maripaludis* occurs by the same pathway used by *Pseudomonas aeruginosa* to make a di-N-acetylated sugar. *J. Bacteriol.* 197 1668–1680. 10.1128/JB.00040-15 25733616 PMC4403654

[B42] Syutkin A. S. van Wolferen M. Surin A. K. Albers S. V. Pyatibratov M. G. Fedorov O. V. (2019). Salt-dependent regulation of archaellins in *Haloarcula marismortui*. *MicrobiologyOpen* 8:e00718. 10.1002/mbo3.718 30270530 PMC6528647

[B43] Szabó Z. Sani M. Groeneveld M. Zolghadr B. Schelert J. Albers S. (2007). Flagellar motility and structure in the hyperthermoacidophilic archaeon *Sulfolobus solfataricus*. *J. Bacteriol.* 189 4305–4309. 10.1128/jb.00042-07 17416662 PMC1913377

[B44] Todor H. Dulmage K. Gillum N. Bain J. R. Muehlbauer M. J. Schmid A. K. (2014). A transcription factor links growth rate and metabolism in the hypersaline adapted archaeon *Halobacterium salinarum*. *Mol. Microbiol.* 93 1172–1182. 10.1111/mmi.12726 25060603

[B45] Tripepi M. You J. Temel S. Önder Ö Brisson D. Pohlschröder M. (2012). N-glycosylation of *Haloferax volcanii* flagellins requires known Agl proteins and is essential for biosynthesis of stable flagella. *J. Bacteriol.* 194 4876–4887. 10.1128/JB.00731-12 22730124 PMC3430349

[B46] VanDyke D. J. Wu J. Logan S. M. Kelly J. F. Mizuno S. Aizawa S. (2009). Identification of genes involved in the assembly and attachment of a novel flagellin N-linked tetrasaccharide important for motility in the archaeon *Methanococcus maripaludis*. *Mol. Microbiol.* 72 633–644. 10.1111/j.1365-2958.2009.06671.x 19400781

[B47] Voisin S. Houliston R. S. Kelly J. Brisson J. R. Watson D. Bardy S. L. (2005). Identification and characterization of the unique N-linked glycan common to the flagellins and S-layer glycoprotein of *Methanococcus voltae*. *J. Biol. Chem.* 280 16586–16593. 10.1074/jbc.m500329200 15723834

[B48] Wieland F. Dompert W. Bernhardt G. Sumper M. (1980). Halobacterial glycoprotein saccharides contain covalently linked sulphate. *FEBS Lett.* 120 110–114. 10.1016/0014-5793(80)81058-1 7439381

[B49] Wieland F. Heitzer R. Schaefer W. (1983). Asparaginylglucose: novel type of carbohydrate linkage. *Proc. Natl. Acad. Sci. U.S.A.* 80 5470–5474. 10.1073/pnas.80.18.5470 16593364 PMC384279

[B50] Wieland F. Paul G. Sumper M. (1985). *Halobacterial flagellins* are sulfated glycoproteins. *J. Biol. Chem.* 260 15180–15185. 3934156

[B51] Wilbanks E. G. Larsen D. J. Neches R. Y. Yao A. I. Wu C. Y. Kjolby R. A. (2012). A workflow for genome-wide mapping of archaeal transcription factors with ChIP-seq. *Nucleic Acids Res.* 40:e74. 10.1093/nar/gks063 22323522 PMC3378898

