# Supplementary material for: N-Glycosylation Is Important for Halobacterium salinarum Archaellin Expression, Archaellum Assembly and Cell Motility
Source: Front Microbiol. 2019 Jun 18;10:1367. doi: 10.3389/fmicb.2019.01367 (PMC6591318; doi:10.3389/fmicb.2019.01367)
Supplement: Supplementary file 1 [file Data_Sheet_1.docx]

**
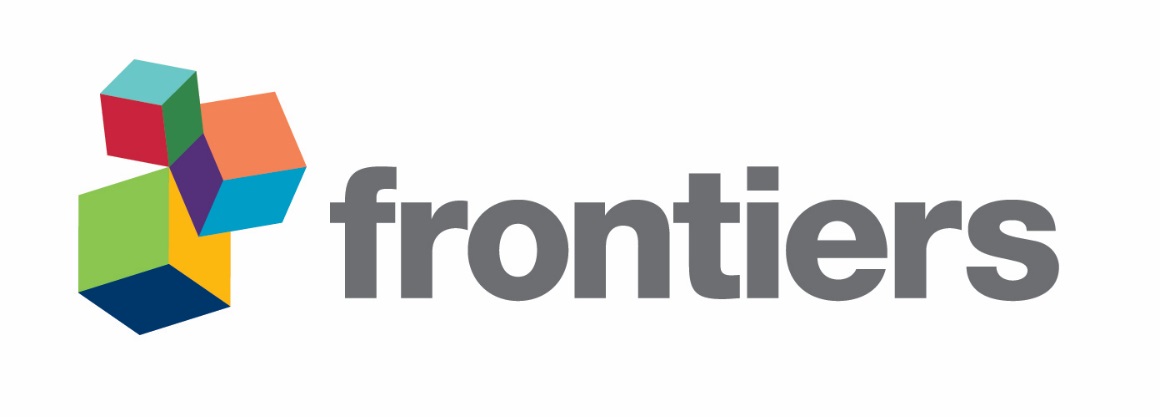
**

**
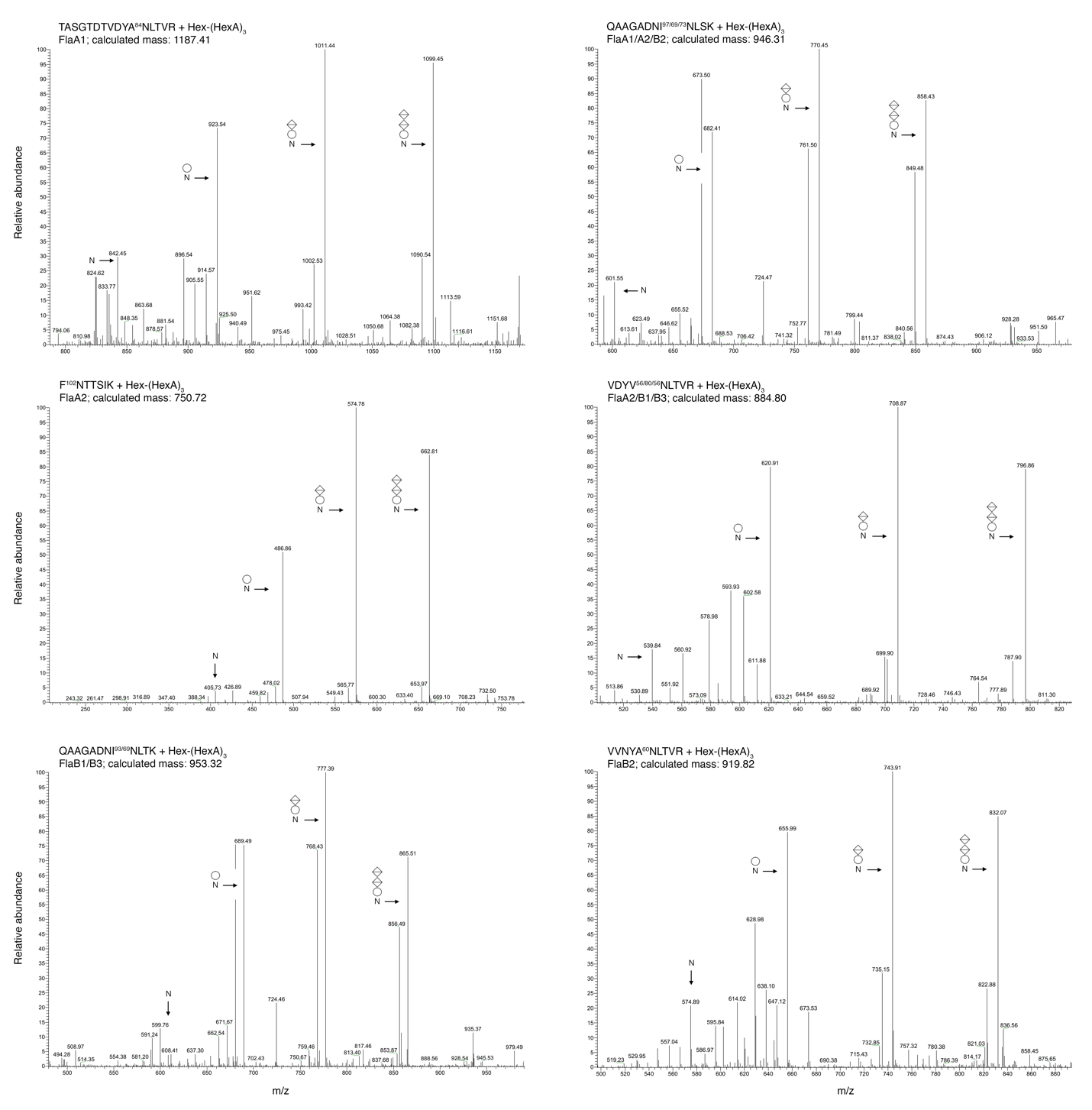
Supplemental Figures**

**
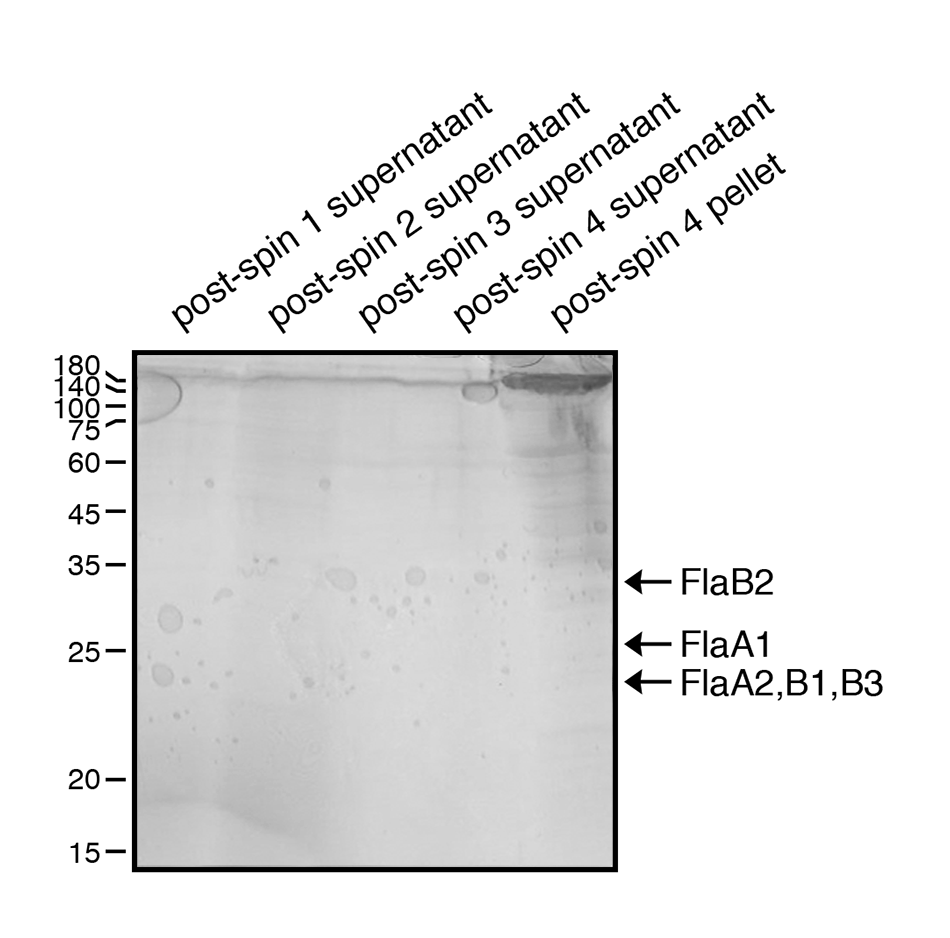
Supplemental Fig. S1** **– Archaellin N-glycosylation revealed by LC-ESI MS/MS.** **A.-E.** MS/MS profiles of the listed tetrasaccharide-charged peptides reveals a fragmentation pattern consistent with the peptide modified by a hexose and a hexose and 1-3 hexuronic acids, as well as the non-modified peptide. In each panel, N corresponds to the modified Asn residue, the circle corresponds to a hexose and the diamonds with a horizontal bar correspond to hexuronic acids. In each panel, the peptide sequence, the position of the modified Asn and the calculated mass of the monoisotopic [M+2H]^2+^ ion peak corresponding to the tetrasaccharide-modified peptide is provided.

**Supplemental Fig. S2 – No archaellins can be enriched from the spent growth medium of the *aglB* deletion strain.** Attempts to enrich *Hbt. salinarum* archaellins from the growth medium of mutant logarithmic and stationary (not shown) phase cultures were made, as described in the Materials and Methods. Aliquots of the indicated fractions collected during isolation were separated by 12% SDS-PAGE and Coomassie-stained. The positions of molecular mass markers are indicated on the left, while the expected positions of the five *Hbt. salinarum* archaellins are indicated on the right.


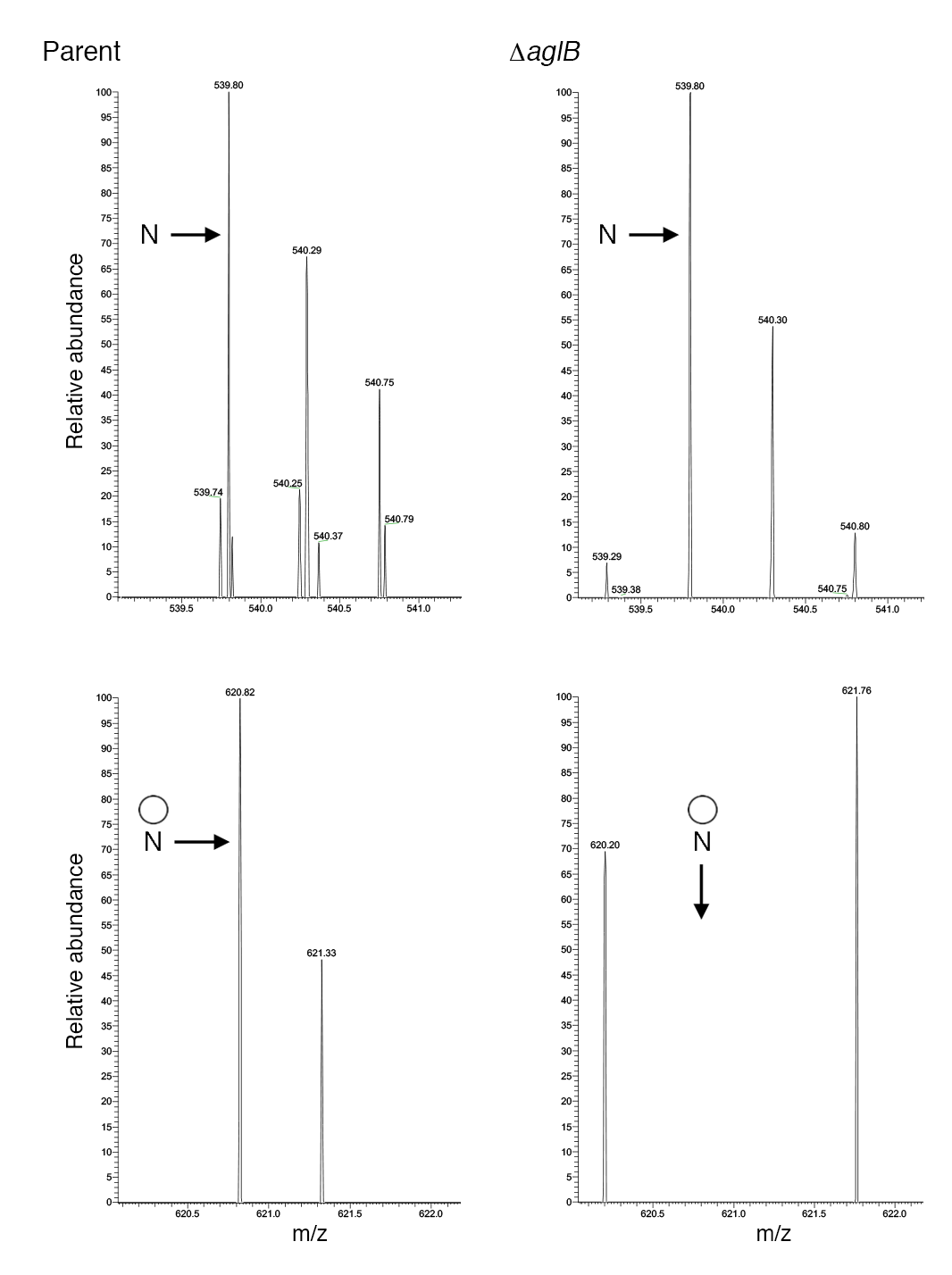


**Supplemental Fig. S3 – N-glycosylation does not occur in the *aglB* deletion strain.** As representatives of what was noted for all archaellin-derived peptides considered in this study, the glycosylation profiles of the FNTTSIK peptide generated from FlaA2 from the parent (left panels) and Δ*aglB* strains (right panels) are presented. The monoisotopic [M+2H]^2+^ ion peaks of the non-modified peptide (upper panels) and the same peptide modified by a hexose in the parent strain (lower left panel) but not in the deletion strain (lower right panel) are shown. The same hexose-modified peptide from the parent strain was further modified by an additional three hexuronic acids (see Supplemental Fig S1); no such modification of the same peptide from the deletion strain was seen (not shown). N corresponds to the modified Asn residue and the circle corresponds to a hexose.

**
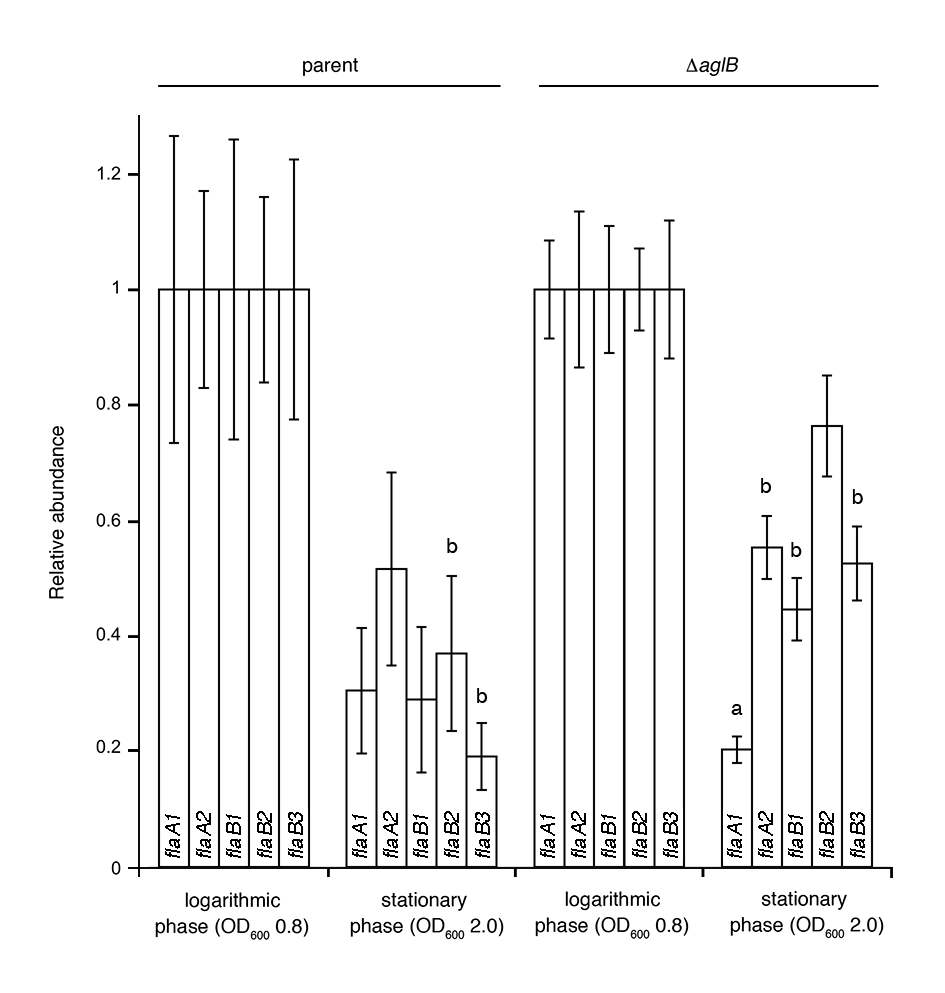
**

**Supplemental Fig. S4 - qRT-PCR reveals reduced transcription of archaellin-encoding mRNA in *Hbt. salinarum* stationary phase cells.** The levels of cDNA, prepared from mRNA isolated from parent or Δ*aglB* strain cells grown to logarithmic or stationary phase (OD_600_ 0.8 or 2.0, respectively), were quantified. The relative abundances of the different archaellin mRNAs are normalized to the value calculated for each strain at logarithmic phase, set as 1.0. The values recorded for the parent strain represent the average of three biological repeats, each comprising eight technical repeats, ± SEM. The values recorded for the mutant strain represent the average of three biological repeats, each comprising four technical repeats, ± SEM. Statistical significance is denoted as follows: a, *p* <0.01; b, *p* <0.05.
